# Highlights from the Second Choosing Wisely Africa conference: a roadmap to value-based cancer care in East Africa (9–10 February 2023, Dar es Salaam, Tanzania)

**DOI:** 10.3332/ecancer.2023.1548

**Published:** 2023-05-10

**Authors:** Rugengamanzi Eulade, Godwin Abdiel Nnko, Jerry Ndumbalo, Nazima Dharsee, Larry O Akoko, Christian Ntizimira, Beda Likonda, Harrison R Chuwa, Salum Lidenge, Verna Vanderpuye, Nazik Hammad, Sikudhani Muya, Fidel Rubagumya

**Affiliations:** 1 Rwanda Cancer Relief, KK 739 Street, Kicukiro, PO Box 4016, Kigali, Rwanda; 2 Department of Clinical Oncology, Muhimbili University of Health and Allied Sciences, PO Box 65001, Dar es Salaam, Tanzania; 3 Department of Oncology, Rwanda Military Hospital, Kanombe, Kicukiro District, Kigali City, PO Box 3377, Kigali, Rwanda; 4 Kingston Health Science Center, Queen’s University, Kingston, ON K7L 3N6, Canada; 5 Ocean Road Cancer Institute, PO Box 3592, Dar es Salaam, Tanzania; 6 Department of Surgery, Muhimbili University of Health and Allied Sciences, PO Box 65001, Dar es Salaam, Tanzania; 7 African Center for Research on End-of-Life Care (ACREOL), KK 349 Street, Kicukiro District, Kigali, Rwanda; 8 Department of Oncology, Bugando Medical Centre, PO Box 1370, Mwanza, Tanzania; 9 Aga Khan Health Service, PO Box 2289, Ocean Road, Dar es Salaam, Tanzania; 10 Korle Bu Teaching Hospital, PO Box 77, Accra, Ghana; 11 Radiation Department, Muhimbili University of Health and Allied Sciences, PO Box 65001, Dar es Salaam, Tanzania; * Rugengamanzi Eulade and Godwin Abdiel Nnko contributed equally

**Keywords:** choosing wisely, Africa, value-based care, cancer, financial toxicity

## Abstract

The ecancer Choosing Wisely conference was held for the second time in Africa in Dar es Salaam, Tanzania, from the 9th to 10th of February 2023. ecancer in collaboration with the Tanzania Oncology Society organised this conference which was attended by more than 150 local and international delegates. During the 2 days of the conference, more than ten speakers from different specialties in the field of oncology gave insights into Choosing Wisely in oncology. Topics from all fields linked to cancer care such as radiation oncology, medical oncology, prevention, oncological surgery, palliative care, patient advocacy, pathology, radiology, clinical trials, research and training were presented to share and bring awareness to professionals in oncology, on how to choose wisely in their approach to their daily practice, based on the available resources, while trying to offer the maximum benefit to the patient. This report, therefore, shares the highlights of this conference.

## Introduction

Approximately 70% of the world’s deaths from cancer occur in low- and-middle-income countries (LMICs) [1]. Tanzania, like other countries in LMICs, is facing a rise in mortality and morbidity l, due to non-communicable diseases. Cancer is ranked as the second most common cause of mortality in the country among other non-communicable diseases [2]. Cancer incidence and mortality are expected to continue rising whereby in 2020 Global cancer data recorded about 40,464: the leading cancers were cervical (25%), breast (10%), and prostate (9%) [3]. Despite the increasing burden of cancer, Tanzania faces many challenges in addressing this issue, including limited access to quality care, a shortage of trained healthcare professionals, little or no clinical trial volume and limited funding for cancer research and treatment [4]. Cancer treatment is evolving rapidly, but most new drugs and technologies are of marginal benefit and not accessible/affordable, in LMICs [5]. Global inequities in cancer care are increasing due to several economic problems that LMICs are facing. Therefore, it has become crucial to consider value-based cancer care, especially in LMICs which considers the quality of care, adverse effects, and cost of care [5].

One of the initiatives that advocate for value-based care is Choosing Wisely. For that reason, the ‘Choosing Wisely Africa (CWA)’ meeting in Tanzania was an important step in addressing the growing burden of cancer in the country and across the continent and also raising awareness of value-based cancer care amongst African oncologists and cancer-treating clinicians. The ‘CWA’ initiative is a vital effort to address the growing burden of cancer in Africa and to ensure that all individuals have access to the care they need. The goal of the meeting was to bring together experts from a range of fields to discuss and find solutions to the challenges facing oncology care in Tanzania. By promoting responsible and effective use of resources, the participants aimed to improve the quality of life for those affected by cancer in Tanzania and across the continent. The meeting was attended, in person, by more than 145 medical personnel. The meeting provided a platform for the exchange of ideas, sustainable networking and the development of a roadmap for future action, in tackling cancer care in Tanzania. This included discussions on the importance of improving access to quality care, the need for greater investment in research and development, leveraging oncology clinical trials for the subregion and the need to build capacity in the healthcare system to better meet the needs of those affected by cancer. The conference was organised by the *e*cancer Global Foundation in partnership with the Tanzania Oncology Society (TOS), Ocean Road Cancer Institute (ORCI), and the Muhimbili University of Health and Allied Sciences (MUHAS) and it was held on 9th and 10th of February 2023 at the Hyatt Regency Hotel in Dar es Salaam, Tanzania.

## The highlights of day 1

The conference was opened with welcoming remarks from Dr Jerry Ndumbalo, a radiation oncologist from ORCI, Tanzania, and President of the TOS, who emphasised the importance of bringing together diverse perspectives to exchange ideas and collaborate on finding solutions to the challenges facing oncology care in Tanzania. He also highlighted the goal of the TOS which is to promote excellence in the comprehensive care of patients with cancer, thereby improving their quality of life, and he finished his remarks by welcoming everyone who came from every side of the world. A series of lectures by various national and international speakers on various facets of epidemiology, education, and research were presented during the conference workshop on day 1 of the event.

### CWA: an initiative that advocates for value-based cancer care

Dr Fidel Rubagumya, a clinical and radiation oncologist from Rwanda Military Hospital, and senior lecturer at the University of Rwanda, talked about the aim and the vision of CWA CWA. Dr Rubagumya gave the background on CWA and how it came about, he further explained how CWA intends to identify low-value, costly, or harmful practices that are frequently used in several African countries. He noted that while there is a growing burden of cancer that is affecting LMICs, including the majority of African nations, there is an urgent need to improve cancer care delivery systems in these nations. He also emphasised the adaptation of guidelines created in high-income countries (HICs) to reflect the healthcare infrastructure, unharmful socio-cultural biases that will not cause harm but increase patient buy-in, and the capabilities of LMICs. Dr Rubagumya finished by highlighting the goal of CWA which is delivering the best care at the lowest cost possible by avoiding financial toxicity to patients, families, and society [6].

### Choosing wisely in educating future African oncologists – the case of the Tanzania Oncology Programme

Dr Nazima Dharsee, the head of the Clinical Oncology Department at MUHAS and the director, Academic and Research Unit at ORCI, spoke about choosing wisely in educating future African oncologists, citing the case of the Tanzanian Oncology Training Programme. She demonstrated the importance of choosing wisely while designing a curriculum that suits and reflect the needs of the African population. She emphasised that the need for in-country training has led to several African universities now creating harmonised curricula as the previous curricula were imported [2]. Dr Dharsee also showed how the Tanzanian oncology training that started in 2010 has impacted the oncology field in the region, impressively, producing several leaders and award-winning oncologists regionally and internationally. The MUHAS/ORCI oncology residency programme has graduated 73 oncologists from 8 African countries since its inception.

### Cancer care in Tanzania

Dr Mark Mseti, clinical oncologist and director of medical services from ORCI, Tanzania, talked about cancer care in Tanzania. He showed how cancer care has improved over the past 10 years, from only 1 hospital with cancer treatment services in 2010 to now, having 14 hospitals in the country, in 2023. However, there has also been a rise in cancer incidence, leading to a significant financial burden on the government as 70% of cancer patients treated in public hospitals in Tanzania are exempted. He also highlighted that there is a big challenge in cancer registries and surveillance, particularly in the validation of data. Furthermore, he highlighted that screening services, particularly in cervical cancer have improved significantly in the country, however, there is still poor coverage and uptake of preventive services such as the human papillomavirus (HPV) vaccine, and a need for intentional ‘Offer at Point of Care’ [7].

### Navigating evidence-based care with limited resources in Africa

Dr Verna Vanderpuye, a consultant oncologist from Korle Bu Teaching Hospital, Ghana, gave a talk about navigating evidence-based care with limited resources in Africa. She started her talk by addressing the lack of funds and grants for studies, poor data recording, poor research culture, poor technology in Africa, absence of research-friendly government or institutional policies, and un-balanced predatory global collaborative research partnerships. As a result, most of the pieces of evidence in the literature are from the HIC which leads to less application of evidence-based medicine in LMICs [8]. This has led to some irrelevant shreds of evidence to the African setting both genetically, culturally and even financially, as most of the current recommendations on cancer therapies, run down the meagre available African budgets and cause financial toxicities to patients and their families. Also, she highlighted gaps in practicing the recommended cancer interventions from HICs in LMICs. She mentioned safety, effectiveness, replicability and scalability. She ended her talk by recommending that African oncologists and policymakers should choose wisely by taking regular course updates including critical appraisal courses, promoting oncology training programmes, setting incentives to promote research activities, and calling for equity in research dissemination globally.

### The role of the workforce in implementing choosing wisely in cancer care

Prof Nazik Hammad, MD, Fellow of the Royal College of Physicians of Canada, Fellow of the American College of Physicians, is a consultant medical oncologist at Queen’s University in Canada. Her talk was about the role of the workforce in implementing choosing wisely in cancer care, she addressed the current reality that by 2040, 67% of annual cancer cases will be in LMICs. With the high cost of cancer treatment, the value can be improved by either increasing quality or decreasing the cost. But there are common quality problems in the practice, those are underuse, overuse and misuse. She demonstrated the three common quality problems through clinical case scenarios which aimed to send a message to the audience to choose wisely in their clinical practices. There is so much harm in the overuse of therapies to patients, relatives, the health system and society, financially. She later emphasised how there is a significant waste of money on medical tourism in Africa to HICs. There is a gap in training the oncology workforce on the economics of health care both in HIC and LMIC, this gap has to be closed by incorporating it into the training programmes.

### Choosing wisely in surgical oncology

Prof Akoko Larry, a consultant surgical oncologist at MUHAS, is currently the only surgical oncologist in Tanzania. He reiterated that choosing oncological procedures, wisely, will result in better outcomes. He further expressed how the shortage of trained surgical oncologists in the region has the potential to escalate unnecessary or unsafe oncological procedures and hence poor patient outcomes. The shortage of surgical oncologists in the region has led to too much medical tourism to India even for simple surgical procedures. He noted that, when he was in training in India, he met a lot of desperate patients from Africa in search of oncosurgery even when palliation was the only choice for them – they held on to the thin thread of hope held out to them, thus further impoverishing their families. To promote patient care, he suggested the selection of a few high-yield oncology conditions and forming disease management teams to promote teamwork and discourage low-value procedures that are currently rampant. He also called upon oncologists to choose wisely by involving surgical oncologists in the management of cancer in what he preferred to call ‘Disease management teams’.

### Choosing wisely in medical oncology

Dr Beda Likonda, a clinical oncologist from Bugando Medical Center in Tanzania, then presented. He started by showing the exponential rise in the cost of cancer care and that the proportion of this expenditure is attributed to unnecessary medical services. That led to 108 recommendations from different institutions, addressing all pillars of the cancer care continuum. Through the American Society of Clinical Oncology (ASCO), ten recommendations have been adopted for medical oncologists, so far. He continued by highlighting a few of the recommendations on how we should choose wisely on investigating patients, he gave an example of low-risk early-stage prostate cancer that a radionuclide bone scan would be unnecessary. On treatment with chemotherapy, oncologists were reminded to choose wisely by not giving chemotherapy to patients with low-performance status (ECOG 3 or 4), and the use of chemotherapy in hormone-positive metastatic breast cancer instead of endocrine therapy. Unwise choosing by oncologists leads to unnecessary toxicities, costs and poor outcomes for our patients.

### Choosing wisely in palliative care in Africa

Dr Christian Ntizimira, a palliative care specialist from The African Center for Research on End-of-Life Care, Rwanda, started his talk on the historical shift in the meaning of death and dying in Africa, where he highlighted the importance of understanding the cultural, spiritual and historical context of African people in providing end-of-life care. Also, he emphasised the need for healthcare providers to be aware of their own biases and assumptions and to engage in ongoing education and training on African cultures and histories [9]. Christian Ntizimira also discussed the concept of Ubuntu (I am because you are or humanity towards others) and its relevance to palliative care to focus on life, humanity, harmony, compassion and dignity. Ubuntu is a philosophy that emphasises the interconnectedness of all people and the importance of community in promoting well-being and healing. Ntizimira argued that Ubuntu can be a valuable framework for providing palliative care in Africa, where community-based care is often the norm. He highlighted the importance of involving patients, families and communities in decision-making and advance care planning, and of providing compassionate, holistic care that addresses the physical, emotional and spiritual needs of patients. He highlighted the importance of collaboration and partnerships between government, non-governmental organisations and other stakeholders to improve the provision of palliative care in Africa. He highlighted successful initiatives and programmes in Rwanda and other African countries that have improved access to palliative care services. He also mentioned adopting versus adapting palliative care in our settings.

## The highlights of day 2

### Choosing cancer medicines wisely in Africa

Dr Harrison R. Chuwa, consultant clinical oncologist and assistant professor at the Aga Khan University and medical director of the Aga Khan Health Service, Tanzania presented about choosing cancer medicines wisely in Africa and started by highlighting the guidelines commonly used in Tanzania. He mentioned the National Cancer Management guidelines, National Comprehensive Cancer Network (NCCN), European Society for Medical Oncology (ESMO) and ASCO Treatment guidelines. He went ahead and elaborated that choosing wisely on cancer medicines in Africa is based on the availability, affordability, accessibility and feasibility of administration of the medicine rather than evidence-based practices. He gave examples of cases of HER2-positive breast cancer requiring dual blockade by Trastuzumab and Pertuzumab [10], according to NCCN guidelines. However, Pertuzumab is not available in the market and even if it was available, it would not be affordable to our patients. He finished by highlighting that choosing cancer medicines wisely is a key strategy in ensuring the optimal management of cancer patients in Africa.

### Choosing wisely to mitigate financial toxicity in cancer care in Africa

Mr Phares Zawadi, a Tanzanian radio-pharmacist with ORCI, Tanzania, gave us a keynote speech about choosing wisely to mitigate financial toxicity in cancer care in Africa, and he said that a diagnosis of cancer leads to a lot of mental, emotional, physical, and financial stress to the patient and family, including using up retirement savings. The impact of finances is shown in non-adherence to medications, and skipping appointments, patients had to cut back on basics like food, clothes and shelter, which can impact their diet and negatively impact their health. Studies show that cancer patients and survivors are more likely to go bankrupt than people without cancer [11]. Financial toxicity has a great impact on cancer treatment, particularly on adherence to treatment and quality of life. To mitigate the financial toxicity, oncologists should choose wisely by avoiding over-prescription.

### Advocacy that impacts patients’ outcomes in Africa – challenges and opportunities

Mr Franklin Mtei, a radiation therapist and patient advocate with ORCI, Tanzania, talked about challenges and opportunities in advocacy that impacts patients. He started by introducing his works in his organisation known as Inspire2Live which deals with patient advocacy and health education to cancer patients regarding cancer and the side effects of treatment. He mainly called upon the use of social media to reach as many patients as possible by creating advocacy and surveillance groups to connect patients. He described how such use of social media can impact patients lives. He raised the issue of the language barrier as the main hurdle of depending on social media, as most of the online content is in English and most Tanzanians do not speak English. It was his view that even a simple intervention like translating the online content to Swahili, can have a huge impact on patients’ lives.

### Emerging global scholars from Africa – is it possible?

Dr Salum Lidenge, a senior lecturer at MUHAS, gave a talk on ‘The role of research in choosing wisely – Emerging scholars in Africa, is it possible?’. Choosing wisely should be based on evidence, and this evidence should be relevant to the targeted population, so research is important in gathering local evidence. Scholars in Africa should focus on conducting research to solve local issues. As one of the recipients of the K43 emerging global leaders award, Dr Salum called upon young scholars to take grant opportunities seriously, as there are many grants for scientists from LMICs. He concluded his talk by answering a question that was part of the heading of his talk that ‘it is possible’.

### Oncology clinical trials in Africa

Prof Ifeoma Okoye, the director of the University of Nigeria Center of Excellence for Clinical Trials (UNNCECT), spoke about oncology clinical trials in Africa, she started her talk by giving statistics that Africa has approximately 15% of the world’s population and only 2% of clinical trials are conducted in Africa. Only 20 of the 54 African countries have hosted clinical trials [12]. She pointed out several factors leading to this underrepresentation of African countries in participation in clinical trials, the factors being patient misconceptions, lack of dedicated research teams, lack of financial and human capacity, and lack of conducive scientific atmosphere (including policy) ethical and regulatory system obstacles. She ended her talk by calling upon African oncologists to unite through the African clinical trial consortium, as a platform for the creation of clinical trial units (CTUs), and quality oversight of clinical trials in Africa. This infrastructure, used in the UK, when they scaled their clinical trial volume, usually assures good clinical practice compliance, which conveys confidence to sponsor companies about the quality of the data that they will be obtaining from such sites. She said the CTU she runs (UNNCECT) is built to match the model of The Clinical Research Centre at University of Cape Town, South Africa. Current efforts are to establish two CTUs in each of the 54 African countries, one at a time. She recommended that Africans should be careful not to allow the Guinea Pig mentality to kill the fledgling industry of clinical trials in our continent and deny our patient population from participating in genomic studies, precision medicine and giving a chance for growth of our indigenous drug development of low hanging candidates with medicinal properties.

Then a recorded video was played of Dr Scott Berry’s speech, the head of the Department of Oncology, Queen’s University, Canada, on how clinical practice guidelines can help clinicians choose wisely. He started his talk with a case presentation of metastatic gastric cancer which has several treatment options on guidelines making it difficult for an oncologist to choose wisely the best regime. He stated that most guidelines do not give direct treatment options and called upon oncologists to choose wisely deciding between the treatment options by an individualised approach based on the patient’s characteristics. He further highlighted the importance of having harmonised guidelines for LMICs to reflect the availability of the recommended therapy.

### Participation of Africa in global, randomised controlled clinical trial – is it beneficial?

This raised the issue of ensuring increased awareness of African scientists, especially oncologists, to be fully trained and empowered to interrogate and choose wisely, which clinical trial studies are beneficial to our African patient population and which are exploitative, taking advantage of our vulnerable population.

### African oncologists’ interaction with pharmaceutical industry

Finally, Dr Nazik Hammad made her second talk which was about African oncologists’ interaction with the pharmaceutical industry, saying that for the past 10 years, the pharmaceutical industry has changed its strategic priorities from antibiotics and anti-retroviral to anticancer drugs. The healthcare system in Sub-Saharan Africa is considered a new market, penetrating this new market with expensive personal gifts, social events and cash payments to doctors is increasingly common in LMICs [13]. Although there are undoubtedly risks involved in industry engagement in LMICs, many programmes with educational, research and clinical values would not occur in these countries without industry support. She argued that oncologists balance the risks and benefits of these relationships to influence patient care.

## Conclusion

The well-attended *e*cancer CWA Tanzania 2023 conference brought together a variety of health professionals and delegates from all over the world together to address the importance of choosing wisely in cancer treatment, research, patient advocacy and surveillance. The speakers emphasised how important it is to factor in feasibility, availability, cultural, economic and social factors when choosing wisely in therapeutic decisions in LMICs. The event addressed the importance of scientists from LMICs performing research and using local data to address local issues in cancer management.

## Conflicts of interest

The meeting was funded by the *e*cancer Global Foundation.

## Funding

None.
